# Palm Kernel Shell as an effective adsorbent for the treatment of heavy metal contaminated water

**DOI:** 10.1038/s41598-019-55099-6

**Published:** 2019-12-12

**Authors:** Rabia Baby, Bullo Saifullah, Mohd Zobir Hussein

**Affiliations:** 1 Materials Synthesis and Characterization Laboratory, Institute of Advanced Technology, Universiti Putra Malaysia, Serdang Selangor, 43400 Malaysia; 2 Education Department, Sukkur IBA University, Sukkur Sindh, 65200 Pakistan

**Keywords:** Environmental impact, Environmental monitoring

## Abstract

Heavy metal contamination in water causes severe adverse effects on human health. Millions of tons of kernel shell are produced as waste from oil palm plantation every year. In this study, palm oil kernel shell (PKS), an agricultural waste is utilized as effective adsorbent for the removal of heavy metals, namely; Cr^6+^, Pb^2+^, Cd^2+^ and Zn^2+^ from water. Different parameters of adsorptions; solution pH, adsorbent dosage, metal ions concentration and contact time were optimized. The PKS was found to be effective in the adsorption of heavy metal ions Cr^6+^, Pb^2+^, Cd^2+^ and Zn^2+^ from water with percentage removal of 98.92%, 99.01%, 84.23% and 83.45%, respectively. The adsorption capacities for Cr^6+^, Pb^2+^, Cd^2+^ and Zn^2+^ were found to be 49.65 mg/g, 43.12 mg/g, 49.62 mg/g and 41.72 mg/g respectively. Kinetics of adsorption process were determined for each metal ion using different kinetic models like the pseudo-first order, pseudo-second order and parabolic diffusion models. For each metal ion the pseudo-second order model fitted well with correlation coefficient, R^2^ = 0.999. Different isotherm models, namely Freundlich and Langmuir were applied for the determination of adsorption interaction between metal ions and PKS. Adsorption capacity was also determined for each of the metal ions. PKS was found to be very effective adsorbent for the treatment of heavy metal contaminated water and short time of two hours is required for maximum adsorption. This is a comprehensive study almost all the parameters of adsorptions were studied in detail. This is a cost effective and greener approach to utilize the agricultural waste without any chemical treatment, making it user friendly adsorbent.

## Introduction

Through the advancement of technology has given us, comforts but at the same industrialization have contributed greatly to the environmental pollution e.g. Water pollution, air pollution and soil pollution. The water and air pollution directly affect the human and soil pollution can contribute to the agricultural and food poisoning, which ultimately contribute to adverse effects to human and other living creatures. Water contamination is a major hazard for living things and human beings and there may be different kinds of contaminants such as bacteria, viruses, organic molecules, dyes and heavy metal ions e.g. Cr^6+^, Pb^2+^, Cd^2+^, Zn^2+^, Ni^2+^, As^3+^ and Hg^2+^ etc. Among all of these water contaminants, heavy metal ions are nonbiodegradable in nature and can accumulate in the human body continuously and may results severe adverse effects such as brain damage, skin diseases, liver damage, kidney failure, anemia, hepatitis, ulcers and are also carcinogenic^1–3^. Heavy metal ions enter the water and environment from different sources, namely industrial wastes, batteries, fertilizers, pesticides, petrochemicals, pharmaceutical, paper and pulp industries etc.^4–6^. The water pollution is making the lives of millions of people at great risks of diseases, illness and even deaths. In addition, water pollution is continuously shortening the availability of drinking water^4,7^. Designing the new valuable methods with easier implementation and cost effective for the purification of water remains a challenge. Different methods are widely applied for the removal of heavy metal ions from the aqueous solutions, namely reverse osmosis, evaporation, calorimetric, ion exchange, precipitation, membrane and coagulation^6,8–10^. Because of the high cost, energy consumption and low concentration of metal ions are problems in above techniques. Agricultural wastes have been exploited as adsorbent for the removal of heavy metal ions from water. They agricultural waste as adsorbents offer many advantages e.g. Low cost, easily available in large quantity and they contain different functional groups like phenolic groups, carbonyl groups, hydroxyl groups, amino, acetamido and sulfhydryl groups etc.^11–13^. These functional groups play a pivotal role in the adsorption of heavy metal ions as they can form complexes and chelates with heavy metal ions. The absorption of heavy metal ions on agricultural adsorbent is termed as absorption, which involves complexations, chelation, chemisorption, diffusion through pores and adsorption on the surface^11–13^. There is a need for the development of user friendly and cost-effective technique for the treatment of heavy metal contaminated water. Every year a lot of agricultural waste is produced from different crops and fields, among them is oil palm waste which is generated in millions of tons every year.

Agricultural waste of oil palm produced millions of tons from Malaysia, Indonesia, Cameron, Africa, China and Nigeria^14^. Malaysia is among the biggest producer and supplier of oil palm with about 4.5 million hectares cultivation and produce about 90 × 10^6^ tons of agricultural waste of oil palm^14^. Palm Kernel shell (PKS) is the sustainable source and usually it is burned to dispose causing greenhouse effect and resulting in a negative impact to the environment. A lot of research efforts are being done to recycle and utilize the agricultural waste for the betterment instead of open burning which making a negative impact to the environment and causing ozone layer depletion^15^. Application of agricultural waste as an adsorbent for the removal of pollutants, especially heavy metal ions from water and that is a cost effective and eco-friendly technique for the treatment of heavy metal ions contaminated water. Different waste materials of plants have been applied for the treatment of heavy metal contaminated water, e.g. Algae, rice husk, corn cob, olive oil by products, livestock waste egg shell, activated carbon, rice husk, sawdust, forest by-products and waste etc.^4,16,17^.

**Figure 1:**
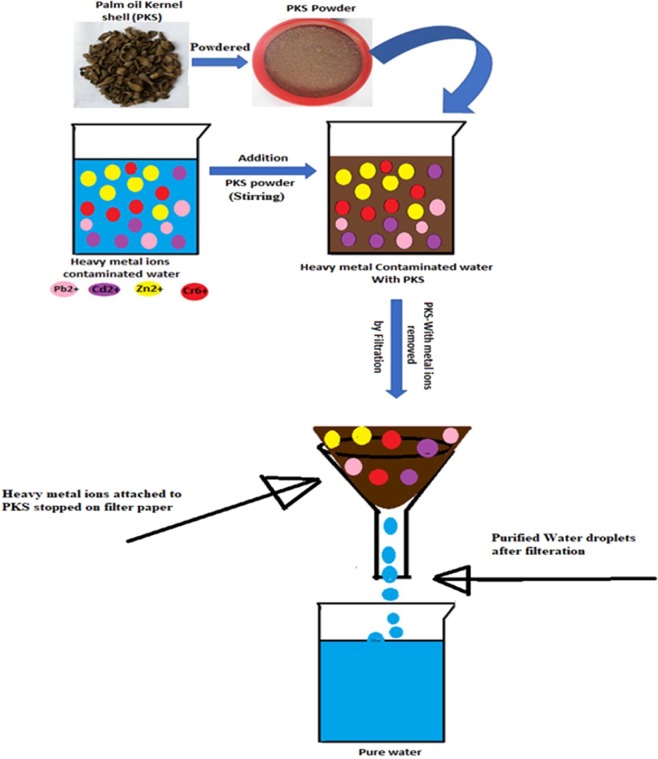
Schematic representation of PKS in the treatment of heavy metal contaminated water.

Palm kernel shell (PKS) of oil palm is useful material to be applied as an adsorbent for the removal of heavy metal ions, as the good quality of organic compounds in it capable of adsorption of metal ions through biosorption mechanisms mentioned above^16,17^. PKS is sustainable agricultural waste produced in millions of tons every year. The disposal of large quantity PKS causes adverse effects to the environment as it disposed-off by burning causing a lot smoke^14,18,19^. In this study, we utilized the PKS as an adsorbent for the treatment of heavy metal contaminated water and the effect of different parameters such as pH of the solution, concentration of metal ions, adsorbent dosage and contact time was determined. In addition to this, different kinetic models and isotherms were used for the determination of the kinetics and sorption process, respectively. PKS was found to be excellent adsorbent for the removal of heavy metal ions from water as it required very short time of 2 hours for the removal of Cr^6+^, Pb^2+^, Cd^2+^ and Zn^2+^ from water with percentage removal of 98.92%, 99.01%, 84.23% and 83.45%, respectively. Figure 1 shows the schematic representation of PKS in the treatment of heavy metal contaminated water.

## Materials and Methods

### Chemicals and reagents

Palm kernel shells (PKS) were collected from Seri Ulu Langat Palm Oil Mill, Dengkil, Selangor Malaysia. The metal ions Cr^6+^, Pb ^2+^, Cd^2+^ and Zn^2+^ solutions, standards of 1000 mg/L, NaOH, HCl were purchased from Sigma Aldrich (St. Louis, MO, USA). The deionized water (18.2 MΩ/cm^−1^) was used during all experiments.

### PKS adsorbent preparation

PKS were washed thoroughly with tape water, followed by washing with deionized water. They were dried in an oven for 48 hours at 70 °C. After that, the PKS were crushed to fine powder by a stable arm grinder and the fine powder was selected for the adsorption studies.

### Experimental set up for batch studies

For the adsorption batch studies, different standard solutions (5 ppm, 10 ppm, 15 ppm, 20 ppm and 25 ppm) of metal ions; Cr^6+^, Pb^2+^, Cd^2+^ and Zn^2+^ were prepared and kept in a refrigerator to maintain the volume and concentration. The adsorption experiments were carried out in flasks 250 mL containing 100 mL metal ion solutions of various concentrations, dosages of PKS powder, pH and contact time. Samples were shaken at previously optimized rate i.e. 200 rpm using a thermostat incubator at room temperature for two hours^4^.

### Characterization

Functional group analysis was carried out using a Fourier transformed infrared (FTIR) spectrometer, Perkin-Elmer 100 series (Waltham, MA, USA). Surface morphology was determined using a Field emission scanning electron microscope (FESEM) JOEL JSM-6400 (Tokyo, Japan). Metal elemental analysis was done using the inductively coupled plasma (ICP), Optical Emission Spectrometer, Optima 2100 DV Perkin Elmer.

## Results and Discussion

### Infrared spectroscopic analysis

**Figure 2:**
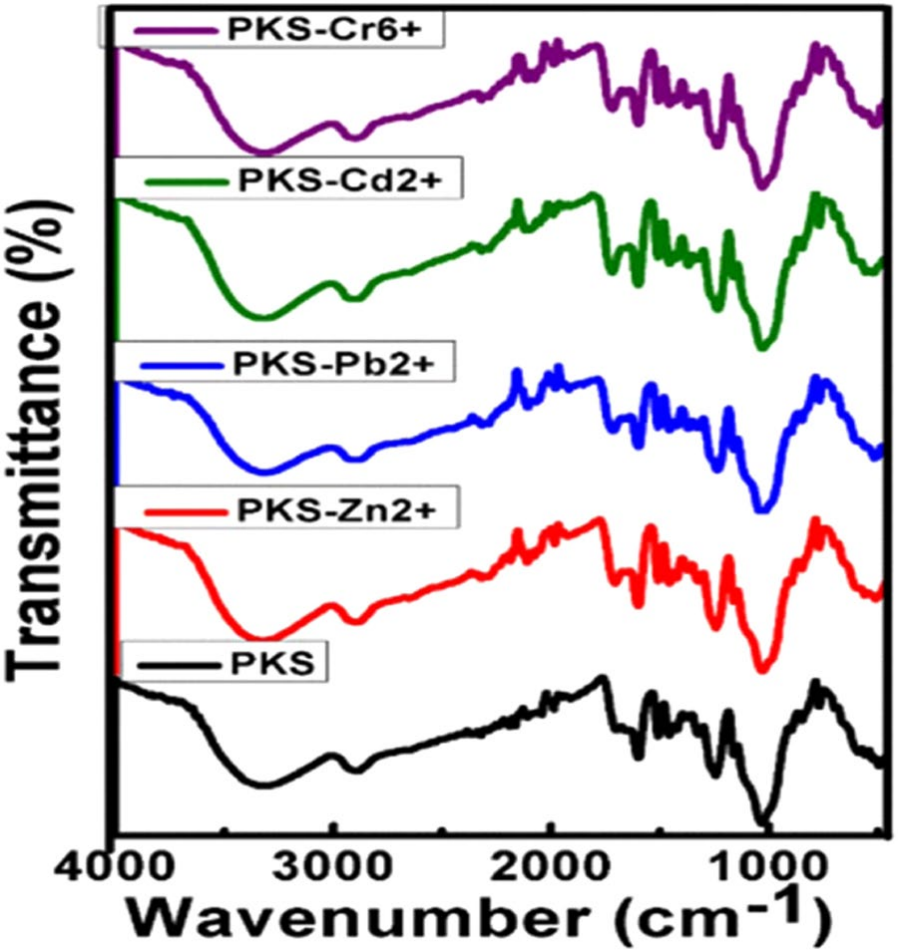
FTIR spectra of PKS after the adsorption process with Cr^6+^ (PKS-Cr^6+^), Zn^2+^ (PKS-Zn^2+^), Cd^2+^ (PKS- Cd^2+^) and Pb^2+^ (PKS- Pb^2+^).

Infrared spectroscopy is a useful technique for the determination of functional groups and any changes take place in the functional groups of any compound. Figure 2 shows the Fourier Transformed infrared (FTIR) spectra of the samples; PKS, PKS-Zn^2+^, PKS-Pb^2+^, PKS-Cd^2+^ and PKS-Cr^6+^. The PKS shows the characteristic band of -OH group at 3338 cm^−1^ and this band has been shifted to the lower wavenumbers, 3313 cm^−1^, 3324 cm^−1^, 3325 cm^−1^ and 319 cm^−1^ after the adsorption of metal ions; PKS-Cr^6+^, PKS-Cd^2+^, PKS-Pb^2+^ and PKS-Zn^2+^, respectively. The PKS after the adsorption of the process showed new twin bands for Cr^6+^ at 2328 cm^−1^ and 2279 cm^−1^, for at Cd^2+^ 2320 cm^−1^ and 2284 cm^−1^, for Pb^2+^ at 2316 cm^−1^ and 2287 cm^−1^ and for at Zn^2+^ 2320 cm^−1^ and 2292 cm^−1^. For PKS alone, these infrared bands are absent, as these new bands appear due the adsorption of metal ions on the carbonyl groups^4,20^. The PKS alone shows the carbonyl band at 1701 cm^−1^ and this carbonyl band has been shifted to higher wavenumber of 1717 cm^−1^, 1718 cm^−1^, 1716 cm^−1^ and 1710 cm^−1^ after the adsorption of Cr^6+^, Cd^2+^, Pb^2+^ and Zn^2+^ ions, respectively. In addition to this, new carbonyl bands were also appeared at about 1655 cm^−1^ after metal ions adsorptions, which was absent in the PKS alone. The infrared band, due to the C-O stretching was appeared at 1043 cm^−1^ and this bands is shifted to the higher values, 1235 cm^−1^ after the adsorption of metal ions^4,20^. The rest of the stretching and bending infrared bands e.g. CH, C-C and C=C etc., were not significantly shifted. There was a significant shift in the infrared bands of O-H, C=O, C-O after the adsorption process. In addition to this, new infrared bands at about 2200–2350 cm−^1^ and at 1650 cm^−1^ were also observed. The shifting and appearance of new infrared bands strongly suggests the successful adsorption of the metal ions on the PKS adsorbents and these results was further confirmed by the elemental analysis. Table 1 shows the detailed infrared bands of PKS before and after the adsorption of metal ions.

**Table 1:** Assignments of infrared bands of PKS before and after the adsorption of metal ions.

Assignment	PKS	PKS-Cr6+	PKS-Cd2+	PKS-Pb2+	PKS-Zn2+
V (O-H) of - COOH)	3338	3313	3324	3315	3319
V (C-H)	2881	2899	2906	2909	2899
CO-M (M=Cr^6+^ Cd^2+^ Pb ^2+^ and Zn^2+^)	—	2328	2320	2316	2320
	—	2279	2284	2287	2292
C=O	1701.	1717	1718	1716	1710
	—	1656	1655	1656	1659
C=C Aliphatic and aromatic	1598	1599	1599	1599	1599
	1507	1507	1508	1507	1507
CH_3_ (assym)	1457	1455	1455	1455	1455
C=C (stretching)	1422	1422	1422	1422	1422
CH_3_ (sym)	1373	1369	1370	1369	1370
	1321	1325	1320	1321	1321
C-O (stretching)	1243	1235	1234	1234	1236
C-C (stretching)	1160	1160	1161	1160	1160
	1031	1028	1028	1031	1029
	897	896	897	897	896
	848	850	848	848	849
CH_2_ (rocking)	767	767	767	767	771

Note: In Table 1 dash line (−) represents the absence of band.

### Field emission scanning electron microscopy analysis

**Figure 3:**
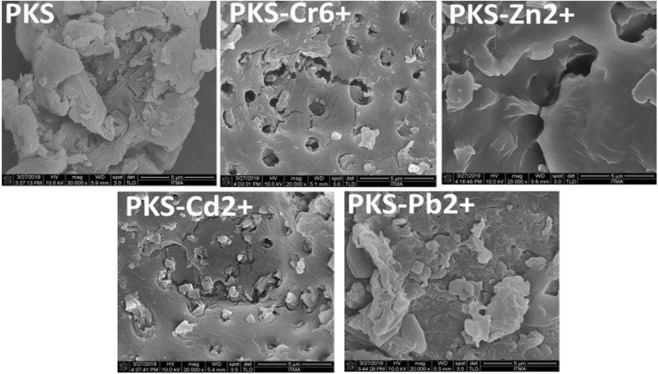
FESEM micrographs of PKS before and after the adsorption process with Cr^6+^ (PKS-Cr^6+^), Zn^2+^ (PKS-Zn^2+^), Cd (PKS-Cd^2+^) and Pb^2+^ (PKS-Pb^2+^).

Figure 3 shows the morphology of the PKS before and after the adsorption of Cr^6+^ ions PKS-Cr^6+^, Zn^2+^ ions (PKS-Zn), Cd^2+^ ions (PKS-Cd^2+)^ and Pb^2+^ ions (PKS-Pb^2+^). Before the adsorption process, PKS morphology is rough with layers, stacking on top of one another and similar morphology has also been reported for PKS our previous study^14^. After the treatment with metal ions e.g. Cr^6+^, Zn^2+^, Cd^2+^ and Pb^2+^ contaminated water, morphology of PKS has changed slightly having a smoother surface as shown in the Fig. 3. The changes in morphology and the formation of pores may possibly be attributed to the process of adsorption via electrostatic and other interactions like chelation, surface adsorption and biosorption. Similar changes in morphology after the adsorption process have also been reported in the literature^21^.

### Effect of pH on adsorption

**Figure 4:**
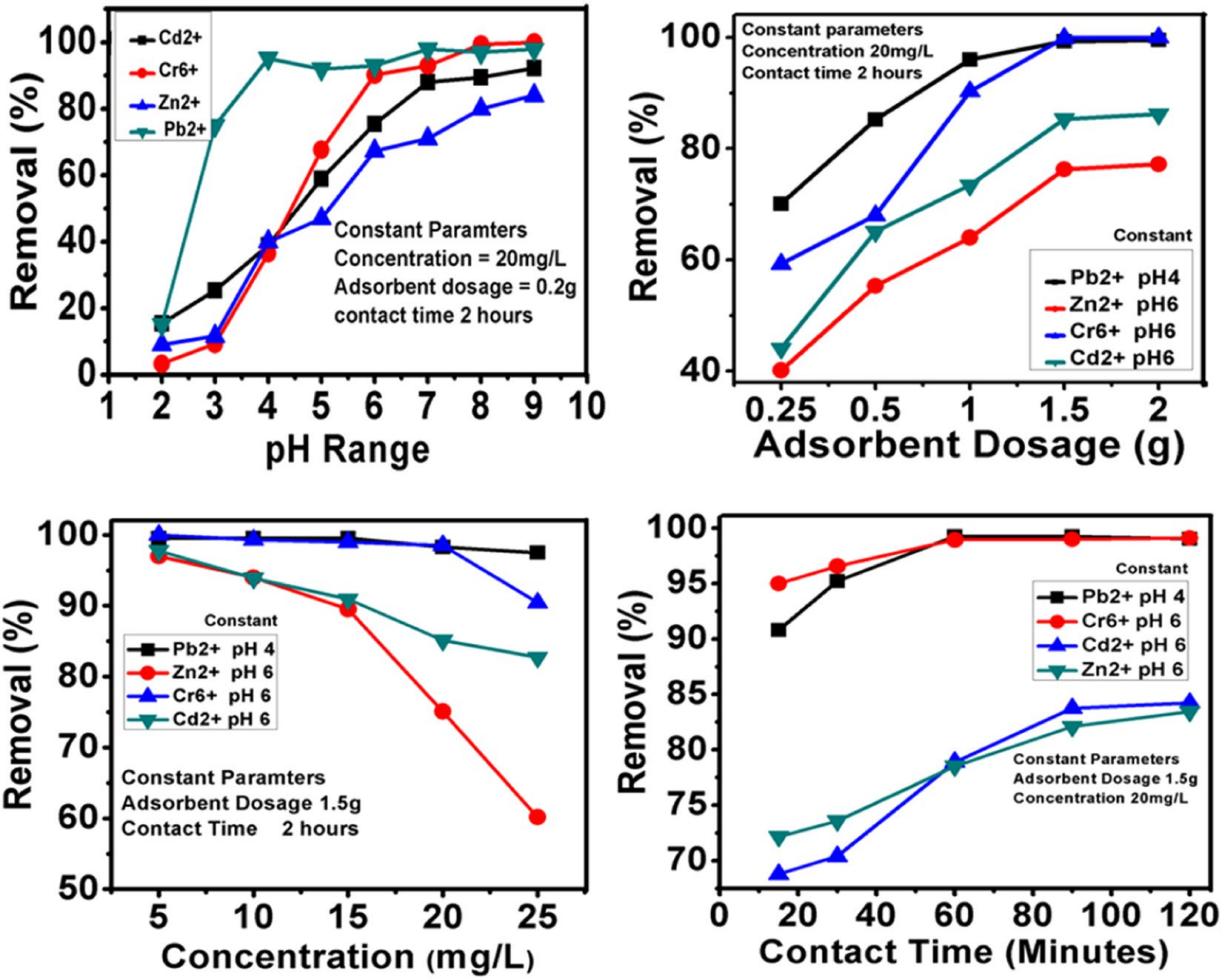
Effect of pH, dosage, concentration and contact time on the adsorption of metal ions; Cr^6+^, Pb ^2+^, Cd^2+^ and Zn^2+^ on the adsorbent (PKS).

In adsorption studies, pH of solution plays pivotal role in the electrostatic interactions between adsorbates and adsorbents^22,23^. In this study, the effect of pH on the removal of heavy metal ions was determined by varying the pH e.g. pH 3, pH 4, pH 5, pH6, pH7, pH8 and pH9 of each of the metal ions; Cr^6+^, Pb^2+^, Cd^2+^ and Zn^2+^. The maximum adsorption was observed in basic pH i.e. above pH7 as shown in Fig. 4. However, in basic conditions, formation precipitation of metal ions as their respective hydroxide can influence the adsorption results, therefore we have selected the maximum adsorption under acidic environment e.g. <pH 7^24–26^. Lead ions showed the highest adsorption of about 95.20% at pH 4 and the remaining three metal ions Cr^6+^, Cd^2+^ and Zn^2+^, pH 6 was found to be the optimum with maximum adsorption of 90.20%, 75.50% and 67.30%, respectively. For the remaining batch experiments, pH 4 for Pb^2+^ ions and pH 6 were selected.

### Effect of dosage of adsorbent

To determine the minimum possible dosage for the maximum adsorption of metal ions, the amount of adsorbent dosage was varied, 0.25 g, 0.50 g, 1 g, 1.50 g and 2 g in 100 mL of 20 ppm metal ion solution. These experiments were conducted under optimized pH conditions for each of the metal ion as discussed in the previous section. Figure 4 illustrates the effect of variation dosage on the adsorption metal ions. The adsorption was increased with the amount of dosage for the Cr^6+^ and Pb ^2+^ ions, which was found to be 59.30% % and 70.12% at an adsorbent dosage of 0.25 g and reached to above almost to about 100% removal of metal ions at 1.5 g of PKS for both these metal ions. The effect of the increase in the amount of PKS on the adsorption of Cd^2+^ and Zn^2+^ was like Cr^6+^ and Pb ^2+^ ions however the overall adsorption of these metals is lower. Adsorption of Cd^2+^ and Zn^2+^ ions were 44.08% and 40.13% at 0.50 g PKS and reached to the maximum value of 86.2% and 77.20% at 2.0 g, respectively. However, there was a very minor difference of 1–3% of adsorption for all the metal ions at 1.5 g and 2 g PKS and therefore for the further batch experiments 1.5 g was used. As can be observed in over trend of adsorption with the adsorbent dosage, the adsorption increases with the increase in dosage and reached to maximum value at 1.5–2 g. These, observations suggest that adsorption is almost directly proportional to the amount of the dosage.

### Effect of the initial metal ions concentration

Figure 4 shows the effect of initial metal ions concentration on PKS adsorption ability. The effect of initial metal ions concentration on the adsorption ability of PKS was determined by varying their concentrations; 5 ppm, 10 pm, 15 ppm, 20 ppm and 25 ppm, under the previously optimized parameters. For Cr^6+^ and Pb^2+^ adsorption trends are almost a straight line with percentage removal of 97.5% and 90.4%, respectively at the highest initial concentration of 25 ppm. However, Cr^+6^ adsorption was decreased from 98.53% (20 ppm initial concentration) to 90.44% (25 ppm initial concentration). The adsorption trends for Cd^2+^ and Zn^2+^ ions showed a continuous decline with the increase in the initial concentration, however the overall adsorption for Cd^2+^ and Zn^2+^ was found to be 82.71% and 60.17%, respectively at 25 ppm initial concentrations. Adsorption of Zn^2+^ at 20 ppm initial concentration was 75.07%, which is still a very high removal percentage. The overall adsorption efficiency decreases with the increase in the initial concentration metal ions, as the adsorption sites in PKS get saturated^27,28^. The maximum adsorption was observed at 20 mg/L and at 25 mg/L the overall percentage of adsorption decreases. As the metal ions concentration increases, the adsorption decreases due to the saturation of sites for chelation or adsorption.

### Effect of contact time on adsorption

One of the important parameters that affect the adsorption process is contact time between the adsorbent and adsorbate^9,29^. A series of experiments for each metal ion solution was conducted by varying the contact time; 15 minutes, 30 minutes, 60 minutes, 90 minutes and 120 minutes and keep the all other optimized parameters constant. Figure 4 also shows the effect of contact time on the adsorption of Pb^2+^, Cr^6+^, Cd^2+^ and Zn^2+^ on the PKS adsorbent. It can be seen in the Fig. 4, that Cr^6+^ and Pb ^2+^ions took 60 minutes to reach the equilibrium with adsorption of about 98%. Cadmium and zinc ions took 90 minutes and 120 minutes to acquire equilibrium with maximum adsorption of about 84% and 83%, respectively.

### Kinetics of adsorption studies

To determine the kinetics of the metal ions adsorption on PKS, different models, namely the pseudo-first order, pseudo-second order and parabolic diffusion were applied.

Pseudo-first order kinetic equation can be written in its linear form as follows

1 $$\mathrm{ln}\,({{\rm{q}}}_{{\rm{e}}}-{{\rm{q}}}_{{\rm{t}}})=\,\mathrm{ln}\,{{\rm{q}}}_{{\rm{e}}}-{{\rm{k}}}_{1}{\rm{t}}$$

where q_e_ and q_t_ is the equilibrium adsorption and adsorption at any time t, respectively and rate constant k_1_ can be determined from the slope by plotting ln(q_e_ − q_t_) versus t.

The linear form of the pseudo-second order can be written as follows

$${\rm{t}}/{{\rm{q}}}_{{\rm{t}}}=1/{{\rm{k}}}_{2}{{\rm{q}}}_{{\rm{e}}}^{2}+{\rm{t}}/{{\rm{q}}}_{{\rm{e}}}$$

The parabolic diffusion equation can be written as

$$1-{{\rm{M}}}_{{\rm{t}}}/{{\rm{M}}}_{{\rm{o}}})/{\rm{t}}={{\rm{Kt}}}^{-0.5}+{\rm{b}}$$

In the above equation, M_o_ and M_t_ are adsorption at time 0 and t, respectively.

**Figure 5:**
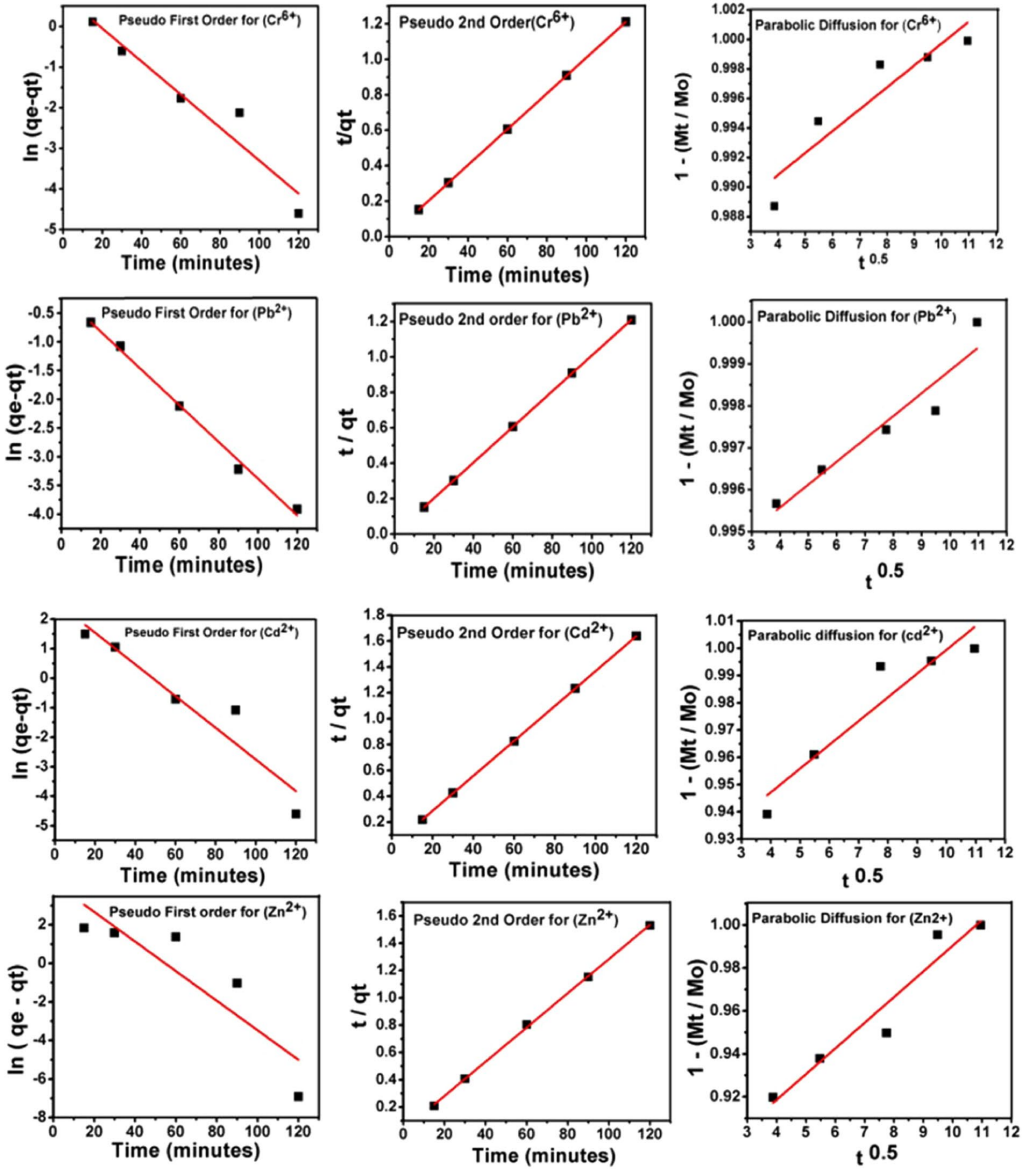
Kinetics adsorption of the metal ions; Cr^6+^, Pb^2+^, Cd^2+^ and Zn^2+^ on to PKS using the pseudo-first order, pseudo-second order and parabolic diffusion models.

Figure 5 shows the kinetics fitting for different models; pseudo-first order, pseudo-second order and parabolic diffusion of all the metal ions; Cr^6+^, Pb^2+^, Cd^2+^ and Zn^2+^ adsorption on the PKS. For each of the metal ion, all three adsorption kinetic models were applied. It was found that the adsorption follows the pseudo-second order process for each of the metal ion, as the correlation coefficient, R^2^ was found to be 0.999 compared to the other two models; pseudo-first order and parabolic diffusion models. Table 2 represents the correlation coefficient, R^2^ value for each model and the value of rate constant, K_2_ for the pseudo-second order. The kinetics of any reaction which follows the Pseudo second order reaction indicates that the adsorption has occurred via chemisorption^30,31^. As discussed in earlier, adsorption involves the chelation, adsorbents functional groups interactions with metal ions. The Pseudo Second order for the adsorption of metal ions by PKS strongly suggests that adsorption occurred via chemisorption.

**Table 2:** Correlation Coefficient (R^2^) and rate constant determined by fitting the data of metal ions adsorption on various kinetic models.

Metal ion	R^2^			Pseudo second order rate constant (K) (mg/minute)
	Pseudo-first order	Pseudo-second order	Parabolic diffusion	
Cr^6+^	0.9111	0.9999	0.8251	1.2 × 10^−5^
Pb^2+^	0.9928	0.9999	0.8855	3.0 × 10^−5^
Cd^2+^	0.8845	0.9999	0.8608	1.2 × 10^−5^
Zn^2+^	0.7286	0.9992	0.9145	2.4 × 10^−5^

### Adsorption isotherms analysis

The isotherm models of Freundlich and Langmuir are realized to determine the mode of adsorption and interaction between the heavy metal ions; Cr^6+^, Pb ^2+^, Cd^2+^ and Zn^2+^ and PKS adsorbent. The equations for Freundlich isotherms (Eq. 1) and Langmuir isotherms (Eq. 4) can be written as follows.

2 $${{\rm{Q}}}_{{\rm{eq}}}={{\rm{k}}}_{{\rm{f}}}\times {{\rm{C}}}_{{\rm{eq}}}\times 1/{\rm{n}}$$

3 $${\rm{Linear}}\,{\rm{form}}\,\mathrm{Log}\,{{\rm{q}}}_{{\rm{e}}}={{\rm{logK}}}_{{\rm{f}}}+1/{\rm{n}}\times {{\rm{logC}}}_{{\rm{e}}}$$

4 $${{\rm{Q}}}_{{\rm{e}}}=({\rm{b}}\,{{\rm{Q}}}_{{\rm{m}}}{{\rm{C}}}_{{\rm{e}}})/(1+{{\rm{bC}}}_{{\rm{e}}})$$

5 $${\rm{Linear}}\,{\rm{form}}\,{{\rm{C}}}_{{\rm{e}}}/{{\rm{q}}}_{{\rm{e}}}=({{\rm{C}}}_{{\rm{e}}}/{{\rm{q}}}_{{\rm{m}}})+1/{{\rm{bq}}}_{{\rm{m}}})$$

where C_e_ and Q_e_ is the equilibrium concentration of metal ions (mg/L) and the amounts of metal ions (mg/g) adsorbed, respectively. QM is the maximum amount of metal ions adsorbed (mg/g) on the PKS, b is a constant and K_f_ and 1/n are Freundlich coefficients.

**Figure 6:**
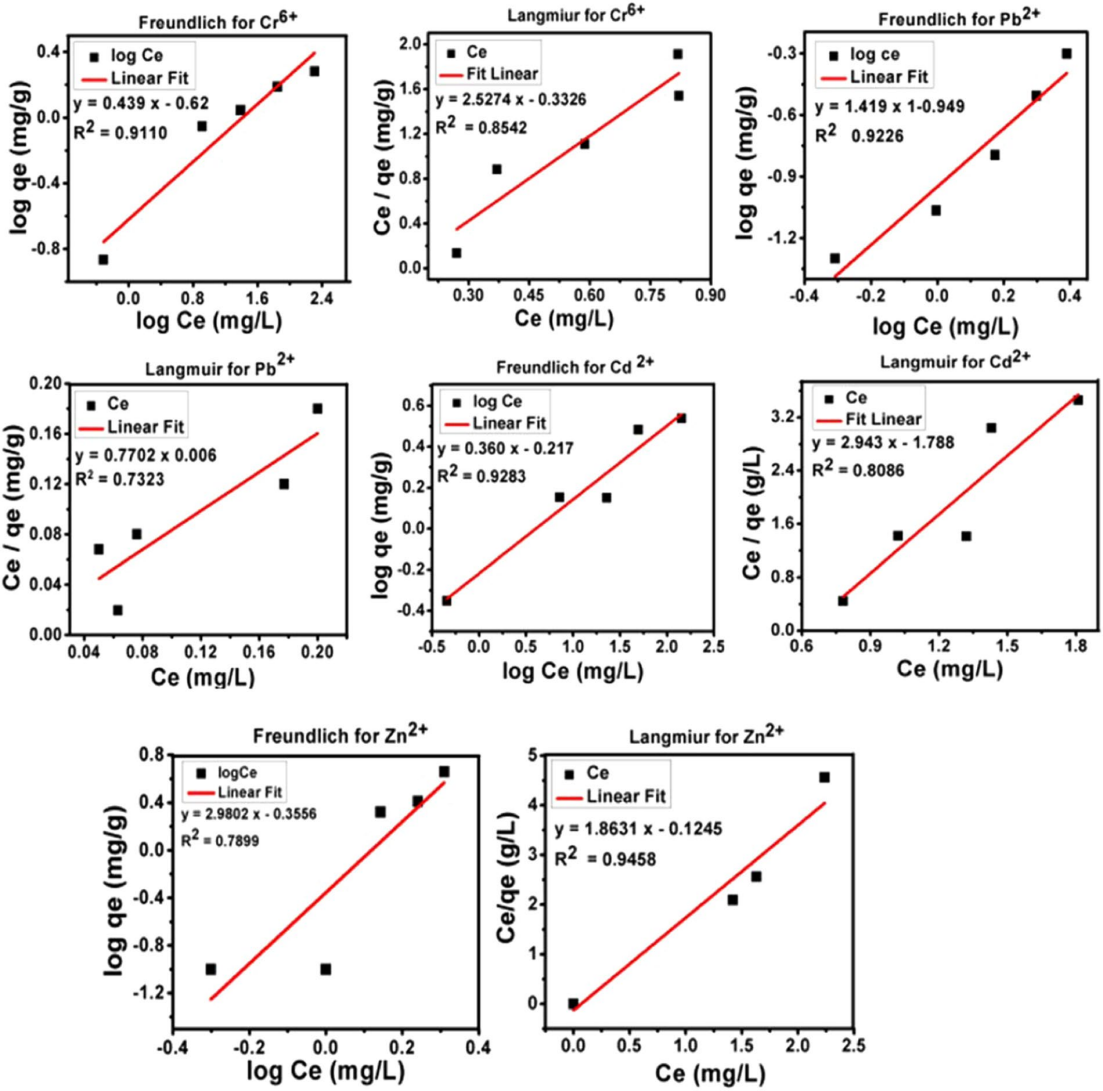
Freundlich and Langmuir isotherms models fitting adsorption for Cr^6+^, Pb ^2+^, Cd^2+^ and Zn^2+^ on PKS.

Figure 6 shows the Freundlich and Langmuir isotherms fitting, and Table 3 shows the correlation coefficient and values of constants for both of isotherm models. The adsorption was found to be in the order of Cr^6+^ > Pb^2+^ > Cd^2+^ > Zn^2+^; however, no significant difference was observed in the adsorption of Cr6+ and Pb 2+. The isotherms study revealed that adsorption fitted well with Freundlich compared to Langmuir model, as the correlation coefficient (**R**^**2**^**)** was found to be higher for Freundlich model which is 0.9 for lead, chromium, cadmium and lower in case of Zn as given in the Table 3. This suggests that the adsorbent (PKS) sites were uniformly spread over the surface and metal ions formed mono layer on the PKS surface. In addition to this, values of **nf** constant for Freundlich model were found to be between 0.1 and 1, suggesting favorable adsorption of metal ions on the PKS surface.

**Table 3:** Langmuir and Freundlich adsorption isotherm values for Cr^6+^, Cd^2+^ Zn^2+^ and Pb^2+^ on PKS.

Metal ion	Langmuir isotherm			Freundlich isotherm		
	q_e_ (mg/g)	b (L/mg)	R^2^	K_f_	n_f_	R^2^
Cr^6+^	49.65	2.57	0.82	0.43	0.62	0.911
Pb^2+^	49.62	1.77	0.23	0.44	0.96	0.943
Cd^2+^	42.12	2.94	0.812	0.36	0.36	0.901
Zn^2+^	41.72	1.86	0.94	2.98	0.35	0.788

### Absorption capacity

**Figure 7:**
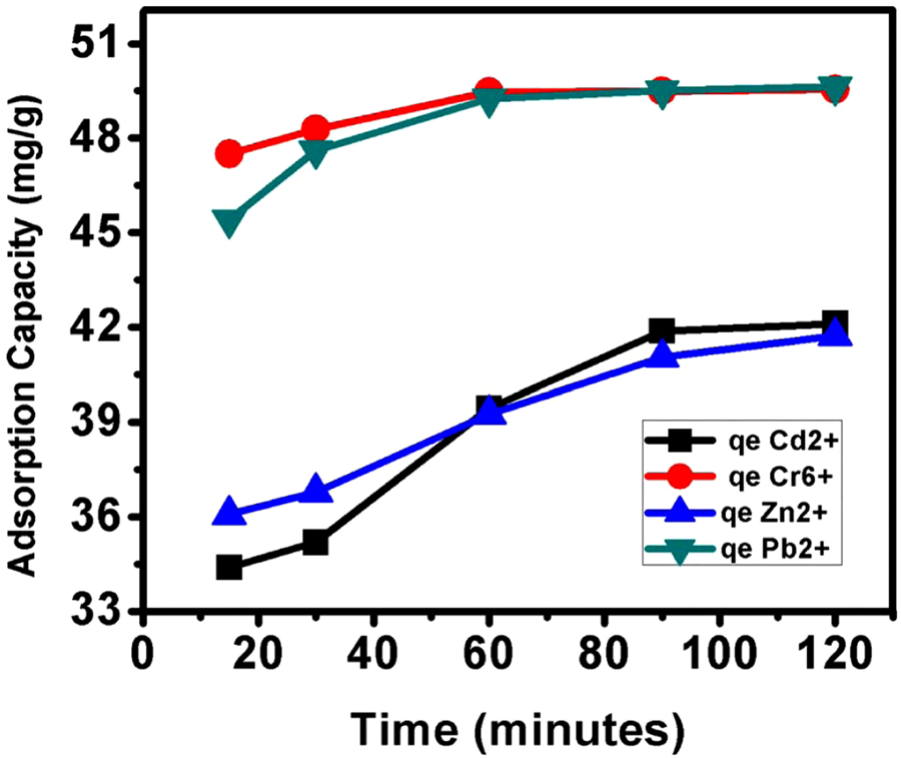
Adsorption capacity (q_e_) of PKS at different time interval for the adsorption of metal ions; Cr^6+^, Pb^2+^, Cd^2+^ and Zn^2+^.

Absorption capacity (qe) of PKS adsorbent was determined by varying contact time and keeping the concentration of metal ions (i.e. 20 ppm/100 mL) and PKS adsorbent dosage (i.e.1.5 g) constant. Figure 7 shows the trends in the adsorption of metal ions on the PKS with respect to time. It can be observed that Cr^6+^ and Pb^2+^ adsorption took place rapidly till 60 minutes, followed by slower adsorption due to saturation of the adsorption sites. The adsorption of Cd^2+^ ions was fast till 90 minutes, followed by a slower one for the last 30 minutes and minor increase in adsorption occurred because saturation of adsorption sites and possibly due to the establishment of the equilibrium. However, for Zn^2+^ ions adsorption was increased gradually in a sharp manner till the first 90 minutes, followed by a relatively rapid adsorption in comparison to the Cd^2+^ and reached to the maximum adsorption at 120 minutes. Table 3 also shows the maximum adsorption capacity qe (mg/g) for each of the metal ions at the longest contact time, 120 minutes.

### Comparative studies

Different bio-adsorbents coconut coir, coconut husk, rape straw powder, Equisetum (EH) and Teucrium (TH) are applied for the removal of heavy metals like Cr^6+^, Pb^2+^, Cd^2+^ and Zn^2+^ from water namely. Table 4 shows the adsorption of capacity (qe) of different adsorbent for the removal of heavy metal ions. The comparative adsorptions as shown in Table 4 revealed that PKS adsorbent is much more efficient compared to the other bio-adsorbents mentioned above.

**Table 4:** The adsorbent Palm Kernel Shell comparison of adsorption capacities (qe) of Zn (II), Cd (II), Cr (IV) and Pb (II) with different bio-adsorbents.

S.No	Metal ions	Adsorbents	q_e_ mg/g
1	Cr^6+^	Palm kernel shell (PKS)	49.65 mg/g
2	Cd^2+^	Palm kernel shell (PKS)	42.12 mg/g
3	Pb^2+^	Palm Kernel Shell (PKS)	49.62 mg/g
4	Zn	Palm Kernel Shell (PKS)	41.72 mg/g
5	Pb	Bacillus cereus (2019)^32^	11.52 mg/g
6	Cd	Bacillus cereus (2019)^32^	10.79 mg/g
7	Cr	Coconut coir (2016)^33^	4.97 mg/g
8	Zn	Rape straw powder (2019)	36.74 mg/g
9	Cr	Coconut husk (2013)^34^	1.96 mg/g
10	Pb	Coconut husk (2013)^34^	2.15 mg/g
11	Cd	Coconut husk (2013)^34^	1.89 mg/g
12	Cd	Equisetum (EH) and Teucrium (TH). (2018)^22^	52.91 mg/gm

## Conclusion

In this study, agricultural waste palm kernel shell was utilized as an adsorbent for the treatment of heavy metals-contaminated water. Toxic heavy metal ions such as Cr^6+^, Pb^2+^, Cd^2+^ and Zn^2+^ were removed from the water using the process of adsorption. Different parameters like solution pH, adsorbent dosage, initial metal ions concentration and contact time were optimized. Under the optimized conditions of all these parameters, about 99% of Cr^6+^ and Pb^2+^ ions and more than 83% of Cd^2+^ and Zn^2+^ ions were removed. The adsorption took 60 minutes for Cr^6+^, Pb^2+^ and 90 minutes and 120 for Cd^2+^ and Zn^2+^, respectively. This is a very short time compared to the other adsorbents which required 6–24 hours. Adsorption capacity of PKS adsorbents for Cr^6+^, Pb^2+^, Cd^2+^ and Zn^2+^ was found to be 49.55 mg/g, 49.64 mg/g, 43.12 mg/g and 41.72 mg/g, respectively. In this PKS was utilized as adsorbent without any pretreatment with acid/base, instead it was just used with water and after drying and grinding it was directly applied for metal ion adsorption. The adsorbent is renewable, cheap and freely available in huge quantity and is produced every year in thousands of tons from oil palm plantations.
